# Metagenomic Analysis of Respiratory Tract DNA Viral Communities in Cystic Fibrosis and Non-Cystic Fibrosis Individuals

**DOI:** 10.1371/journal.pone.0007370

**Published:** 2009-10-09

**Authors:** Dana Willner, Mike Furlan, Matthew Haynes, Robert Schmieder, Florent E. Angly, Joas Silva, Sassan Tammadoni, Bahador Nosrat, Douglas Conrad, Forest Rohwer

**Affiliations:** 1 Department of Biology, San Diego State University, San Diego, California, United States of America; 2 Department of Computational Sciences, San Diego State University, San Diego, California, United States of America; 3 Department of Medicine, University of California San Diego, La Jolla, California, United States of America; 4 San Diego VA Healthcare System, San Diego, California, United States of America; 5 Center for Microbial Sciences, San Diego, California, United States of America; Oregon Health & Science University, United States of America

## Abstract

The human respiratory tract is constantly exposed to a wide variety of viruses, microbes and inorganic particulates from environmental air, water and food. Physical characteristics of inhaled particles and airway mucosal immunity determine which viruses and microbes will persist in the airways. Here we present the first metagenomic study of DNA viral communities in the airways of diseased and non-diseased individuals. We obtained sequences from sputum DNA viral communities in 5 individuals with cystic fibrosis (CF) and 5 individuals without the disease. Overall, diversity of viruses in the airways was low, with an average richness of 175 distinct viral genotypes. The majority of viral diversity was uncharacterized. CF phage communities were highly similar to each other, whereas Non-CF individuals had more distinct phage communities, which may reflect organisms in inhaled air. CF eukaryotic viral communities were dominated by a few viruses, including human herpesviruses and retroviruses. Functional metagenomics showed that all Non-CF viromes were similar, and that CF viromes were enriched in aromatic amino acid metabolism. The CF metagenomes occupied two different metabolic states, probably reflecting different disease states. There was one outlying CF virome which was characterized by an over-representation of Guanosine-5′-triphosphate,3′-diphosphate pyrophosphatase, an enzyme involved in the bacterial stringent response. Unique environments like the CF airway can drive functional adaptations, leading to shifts in metabolic profiles. These results have important clinical implications for CF, indicating that therapeutic measures may be more effective if used to change the respiratory environment, as opposed to shifting the taxonomic composition of resident microbiota.

## Introduction

Each day the human respiratory tract comes into contact with billions of airborne particles, including viruses, microbes and allergens [1]. Particle size and the local airway host immune response determine which inhaled viruses and particles will adhere to epithelial surfaces and persist in the airways [1], [2]. The lungs and lower respiratory tract have generally been considered sterile in the absence of respiratory disease although very little is known about the microbiota of the upper and lower airways of non-diseased individuals. Microbes and viruses, including phage, have been implicated in chronic pulmonary diseases, such as chronic obstructive pulmonary disease (COPD), asthma, and cystic fibrosis (CF) [3]–[8]. However, most of this work has been performed using standard microbial cultures and PCR-based studies, which provide an incomplete picture of the airway microbiota and little opportunity for viral discovery compared to metagenomic techniques.

Metagenomics is a powerful tool for viral and microbial community characterization since nucleic acids are isolated directly from environmental samples and sequenced, requiring no culturing, cloning, or *a priori* knowledge of what viruses may be present. Viruses are the most numerous and diverse biological entities on Earth, and metagenomics has been used extensively to describe viral communities in marine ecosystems [9]–[12]. The first metagenomic studies of the human microbiome were of viruses in blood, feces, and the lungs, and went far to describe previously unexplored viral communities [13]–[17]. Recent metagenomic studies of the human microbiome have largely focused on microbial populations, predominantly in the gut and the surface of the skin [18]–[21].

Cystic fibrosis is an autosomal recessive genetic disease caused by a mutation in the cystic fibrosis transmembrane conductance regulator protein (CFTR), a gated ion channel [22], [23]. CF affects paranasal sinuses as well as the lower respiratory, hepatobiliary, pancreatic and lower gastro-intestinal tracts [23]. The current median age of survival for individuals with CF is approximately 38 years. Over 80% of CF mortalities are attributable to respiratory failure from chronic bacterial infections of the lungs, most commonly caused by *Pseudomonas aeruginosa*, *Staphylococcus aureus*, and *Burkholderia cepacia* [4], [24]. Individuals with CF have impaired mucociliary clearance (MCC) which results in airway mucus plugging [2] [25] [2]. This creates hypoxic microenvironments, forcing invasive microbial species to adapt [2]. This unique airway environment is believed to increase viral replication and susceptibility to viral infections in individuals with CF [8], [22]. Expectorated sputum provides a sample of airway secretions from the proximal airways. Sputum also contains material from the entire respiratory tract including airway mucus, cellular debris, DNA, and degraded proteins as well as microbes, their associated phage, and eukaryotic viruses [26], [27].

Here we report the first metagenomic study of airway DNA viral communities using sputum samples from both cystic fibrosis and Non-cystic fibrosis (Non-CF) individuals, including the spouse of an individual with CF and an individual with mild asthma. Viral communities from Non-CF volunteers were characterized and compared to viromes of individuals with cystic fibrosis to determine if there is a core respiratory tract virome in non-diseased individuals. Metabolic profiles inferred from metagenomic sequences were distinctly different between Non-CF and CF viromes. Our results indicate that regardless of the presence or absence of shared taxa, a core set of metabolic functions defines the non-diseased and diseased respiratory tract DNA viromes.

## Results and Discussion

### Phage taxonomy reflects airway pathology

**Figure 1:**
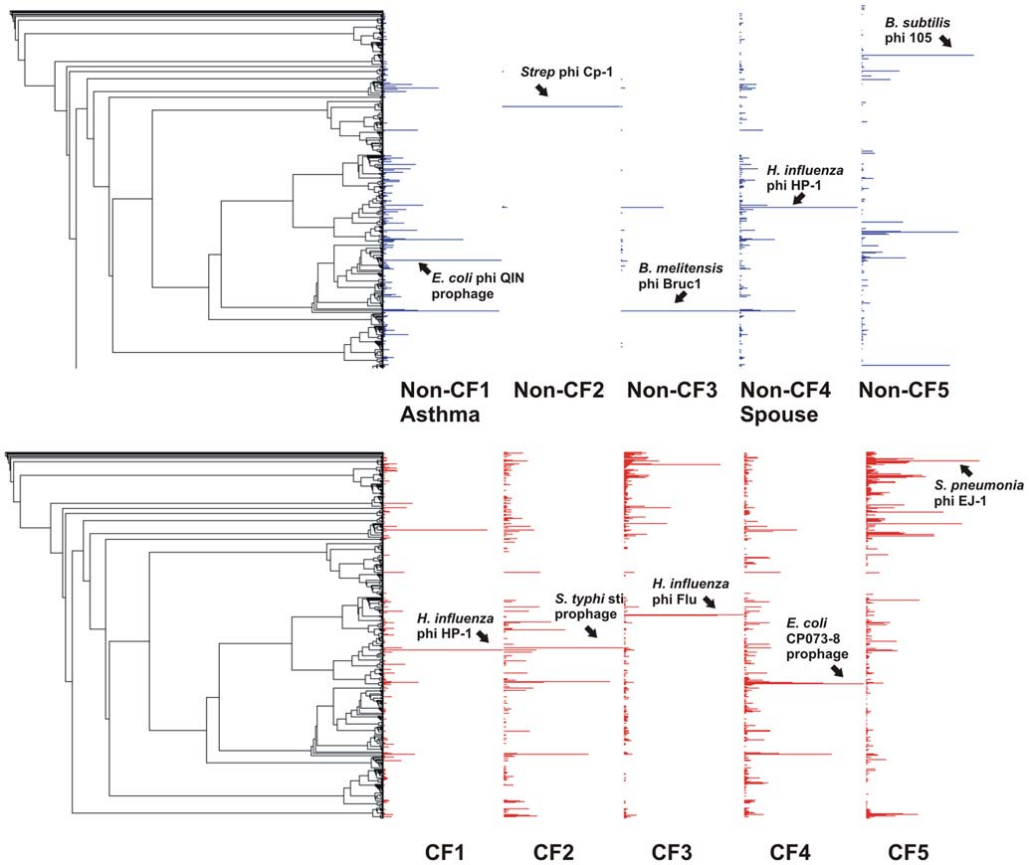
Mapping of best tBLASTx hits to the phage proteomic tree by percentage for Non-CF (A) and CF (B) viromes. The phage genome with the highest percentage of hits (normalized to the length of the genome) is labeled for each virome.

In all metagenomes, the majority of sequences (>90%) were unknown when compared to the non-redundant database using BLASTn (Table S1), which is comparable to the percentage of unknown sequences in other environmental viromes [9], [12], [28]. CF viromes had more tBLASTx similarities to phage genomes overall than Non-CF viromes, and were similar to a wider range of phage (Figure 1, Table S2). The tBLASTx analysis identified a core set of 19 phage genomes which had similarities to sequences in all metagenomes (Table S3). An additional 12 genomes had significant similarities to viromes from all CF individuals but none of the Non-CF individuals. This suggests a core set of phage characteristic of the human respiratory tract, and an additional core group in CF individuals. A few phage genomes appeared to dominated the Non-CF2, Non-CF3, and Non-CF5 viromes when tBLASTx similarities to phage genomes were plotted against the Phage Proteomic Tree (Figure 1). Over 90% of tBLASTx hits to phage in Non-CF2 were to *Streptococcus* phage Cp-1, and 80% of tBLASTx similaritites in Non-CF3 were attributable to two phage, *Haemophilus influenza* phage HP-1 and *Brucella melitensis* 16 M BrucI prophage. The large relative abundance of these phage may reflect their prevalence in inhaled air, since environmental air has been shown to contain diverse bacterial communities [29].

**Figure 2:**
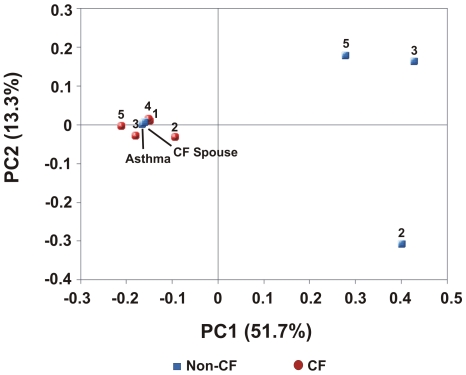
Principal components analysis (PCA) of respiratory tract viromes based on phage taxonomic composition. Non-CF metagenomes are shown in blue and CF metagenomes are shown in red. Inputs to PCA were normalized percentages of best tBLASTx hits to completely sequenced phage genomes. Non-CF1Asthma and Non-CF4Spouse cluster with the CF metagenomes.

The phage profiles of Non-CF1Asthma and Non-CF4Spouse were more similar to those of CF individuals than to other Non-CF individuals. This likeness was confirmed by PCA (Figure 2). Non-CF1Asthma and Non-CF4Spouse had values for the first and second principal components which were nearly identical to those of the CF metagenomes. The other Non-CF metagenomes had more random distribution of phage genotypes and did not appear to cluster on the PCA graph. More specifically, Non-CF2, NonCF3, and Non-CF5 all had positive values for the first principal component (0.40, 0.42, and 0.27 respectively) while all other metagenomes had negative values. This was driven by a large positive loading of the first principal component by the *Streptococcus* phage Cp-1, which segregated Non-CF2, and negative loadings on the set of phage genomes shared by Non-CF1Asthma, Non-CF4Spouse and the CF metagenomes. Additionally, the second principal component was positively loaded by the *Brucella melitensis 16 M* phi Bruc1 prophage genome which was nearly absent in Non-CF2, giving a negative value of the second principal component for Non-CF2.

These results indicate that the sputum phage community in Non-CF individuals appears to represent a random, transient sampling of the exterior environment. In CF individuals, phage communities are driven by airway pathology, and correspond to a shared internal respiratory environment. The phage community in the Non-CF4Spouse virome reflects a continuous sampling of CF-associated phage via a shared external environment. Common phage taxonomy in CF individuals and Non-CF1Asthma occurs because of shared respiratory pathology (i.e., similar internal environments). Both CF and asthma are conditions marked by impaired mucociliary clearance (MCC) [2], [25], [30]. MCC is slowed in asthma, leading to increased retention of microbes and hence their phage [30]. In CF, mucus is extremely viscous and stagnant, forming obstructive plugs, and creating hypoxic microenvironments that serve as scaffolds for bacterial biofilm formation [2], [25]. Therefore, in both asthma and CF, phage communities are derived from microbes which persist in the airways for longer periods of time than in healthy individuals.

### Inferred host ranges for respiratory tract phage

**Figure 3:**
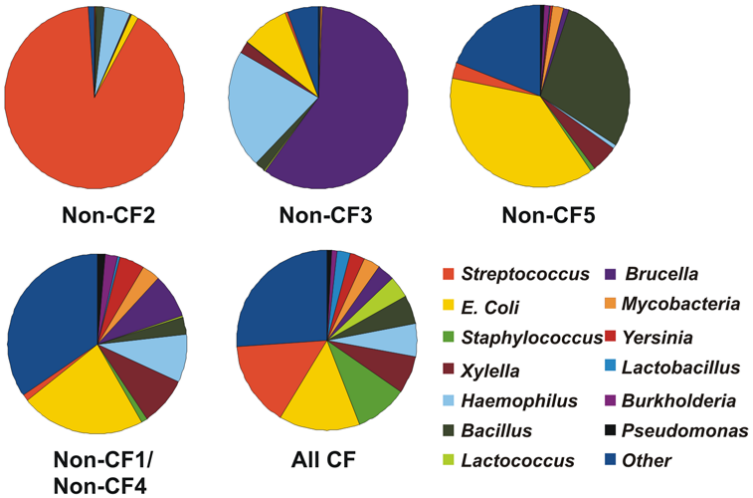
Putative host range for phage communities in respiratory tract viromes. Host range was inferred from normalized best tBLASTx hits to phage genomes. Host ranges for CF viromes and for Non-CF1Asthma and Non-CF4Spouse were not statistically significantly different as determined by XIPE and were combined.

The putative microbial host range of respiratory tract phage reflected a few dominant but distinct phage in Non-CF2, Non-CF3, and Non-CF5 (Figure 3).Host ranges of Non-CF1Asthma and Non-CF4Spouse were highly similar to those of the CF phage communities, but were under-represented in *Streptococcus* and *Staphylococcus* phage. The higher abundance of *Staphylococcus* phage in CF is consistent with the increased induction of *Staphylococcus* prophage by antibiotics in CF individuals, as shown by previous studies [31]. *P. aeruginosa* was cultured from the sputum of all CF participants, yet *Pseudomonas* phage were not abundant in the metagenomes. *Pseudomonas* phage may be of novel types not closely related to those in the database, making them undetectable by tBLASTx. Even if known phage are present, infections of *Pseudomonas* in CF may be unsuccessful, since phage may not be able to penetrate the biofilm to access susceptible microbial hosts [32]. Alternatively, *P. aeruginosa* may not be as abundant in the CF airway as indicated by culturing, an idea supported by 16S rDNA and Terminal Restriction Fragment Polymorphism (T-RFLP) analysis of bacteria in CF sputum and bronchoalveolar lavage fluid [33]–[35]. T-RFLP uses fluorescently labeled 5′ PCR primers coupled with restriction digests to allow for rapid profiling of unknown microbial communities, providing a less biased picture of microbial diversity than culture-based studies [35].

### Diversity of respiratory tract viruses

There were approximately 175 unique species of DNA viruses in respiratory tract viral communities (Table 1). There were no significant differences in the estimated number of species between CF and Non-CF viromes. Diversity estimates were based on sequence assemblies and PHACCs, so all metagenomic sequences were used, not just those with BLAST similarities to viral databases [36]. The estimated number of DNA viral species has been reported to be as low as 1440 in hot springs, and as high as 129,000 in the open ocean [9], [37]. In comparison with other environmental viromes, the respiratory tract viromes had low species richness. Similarly, Rogers et al. [34] found low diversity of Bacteria in CF sputum using T-RFLP analysis. Low species richness probably results from physical and biological barriers to microbial and viral persistence, including both MCC as well as innate and adaptive immunity [2], [38]. Richness may be further depressed in CF individuals because of antibiotic therapies and the metabolic adaptations required for microbial and viral survival in the unique microenvironment of the CF airway [26], [27].

**Table 1:** Diversity estimates for human respiratory tract DNA viromes.

Sample	Species Richness	Evenness	Shannon Index
NonCF1Asthma	164	0.89	4.52
NonCF2	156	0.95	4.81
NonCF3	113	0.94	4.45
NonCF4Spouse	187	0.94	4.92
NonCF5	594	0.86	5.46
**NonCF Mean**	**243**	**0.92**	**4.83**
CF1	69	0.85	3.85
CF2	154	0.86	4.34
CF3	104	0.8	4.32
CF4	121	0.92	4.42
CF5	75	0.84	3.91
**CF Mean**	**105**	**0.85**	**4.17**
**Overall Mean**	**174**	**0.89**	**4.5**

Repeated sets of 10000 random sequences were retrieved from each metagenome and assembled to obtain contig spectra. Diversity modeling based on contig spectra was performed with PHACCs, using a logarithmic model and an average genome size of 50 kb.

Cross-BLASTn analysis showed that CF viromes shared more sequences with each other than Non-CF viromes. Sequences from each metagenome were compared pairwise to all other metagenomes using BLASTn to identify shared sequences as explained in Methods [39]. The majority of the common CF sequences were not found in any Non-CF metagneomes. Sequential BLAST analysis identified 31,413 sequences common to all CF viromes, and 12,824 of these did not appear in any of the Non-CF viromes. Non-CF viromes shared 11,995 sequences, and 330 could not be found in any CF virome. Both the larger group of shared and unique sequences in CF metagenomes suggests that CF viral communities are more similar than Non-CF communities.

### Taxonomy of eukaryotic viruses

**Figure 4:**
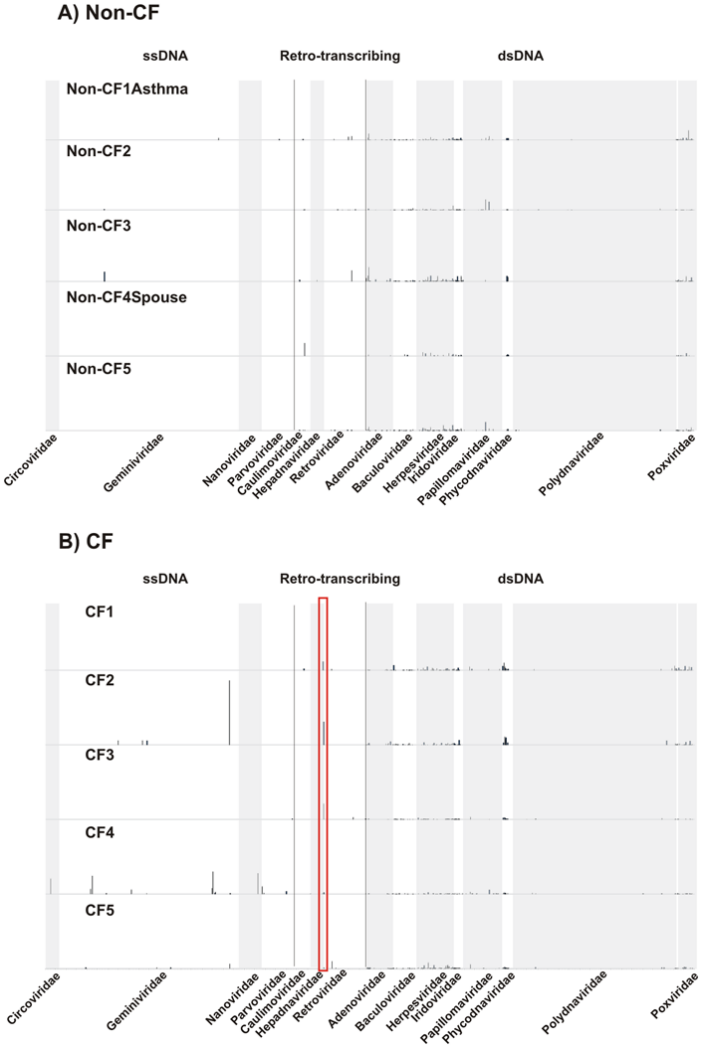
Distribution of normalized best tBLASTx hits to DNA and Retro-transcribing eukaryotic viruses in Non-CF(A) and CF(B) individuals. Reticuloendotheliosis virus is indicated by the red rectangle in (B).

Eukaryotic DNA viral communities in CF individuals were dominated by a few viral genomes which were highly variable in their abundances. Non-CF individuals shared numerous eukaryotic viruses with more even abundances, suggestive of a core virome (Figure 4A). All CF metagenomes had similarities (>1%) to Reticuloendotheliosis virus (Figure S1) and other retro-transcribing viruses (Figure 4B). We confirmed bioinformatically that similarities to retroviruses were not actually similarities to the human genome, therefore, we assume that retroviruses must have been present in the metagenomes as DNA intermediates. indicating that retroviruses may establish persistent infections in the airways, and could be useful therapeutic vectors for CF as previously suggested [40]. CF viromes also shared several human herpesviruses (HHV) including Epstein-Barr virus (HHV-4), HHV-6B, and HHV-8P. Infection with Epstein-Barr virus in adolescent CF patients has been linked to exacerbations and poor clinical outcomes, and has also been observed in adults [41].

CF2 and CF4 had many similarities to Geminiviruses and Nanoviruses, single-stranded DNA viruses of plants (Figure 4B). However, these similarities were concentrated at one location in the genome, the coding sequence for the replication initiator (Rep) protein. Specifically, they were localized to the WalkerA and WalkerB motifs of Rep which correspond to an ATP-binding domain in the translated protein [42]. ATP-binding motifs are common to Rep proteins from a variety of viruses, including Geminiviruses, Nanoviruses, Circoviruses, Parvoviruses, and phage [42]. Therefore, tBLASTx similarities to specific Rep motifs indicate the presence of a virus, but not specifically a Gemini- or Nanovirus.

Non-CF viromes had similarities to fewer unique viral genomes, that is, there were fewer genomes with tBLASTx hits only in one virome (Table S4). Non-CF3 had significant similarities to a Geminivirus, but all hits were to the WalkerA and Walker B motifs of the Rep protein. Human papillomavirus Type 34 comprised over 5% of tBLASTx hits in both Non-CF2 and Non-CF5, and Non-CF2 also had many similarities to Human papillomavirus type 71. Human papillomaviruses have been been detected previously in the respiratory tract and are commonly found in tumors in the lungs and the oropharynx [43]–[45].

The majority of viral species found in Non-CF viromes were from a core set of 20 viral genomes, which were shared by all metagenomes (Table S5; Figure S2). These included a mammalian adenovirus (Bovine adenovirus A), eight mammalian herpesviruses, and three poxviruses. Adenoviruses and herpesviruses have been detected in the airways of both CF and Non-CF individuals, and tBLASTx similarities to non-human viruses represent related undiscovered human variants [8]. Several other viruses, such as algal and insect viruses, were shared among all metagenomes, but similarities to these viruses were largely concentrated in one area of the genome. Since metagenomics allows direct sequencing of environmental DNA, metagenomic techniques often isolate novel viruses and microbes. The hallmark of a novel viral genotype is a large concentration of sequences in one discrete region of a previously sequenced genome. Therefore, these results suggest the presence of a novel virus common to all individuals which cannot be identified using database similarities, analogous to viruses detected in human blood [15].

**Figure 5:**
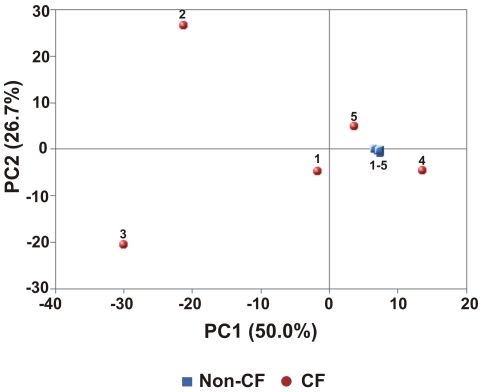
Principal components analysis based on best tBLASTx hits to 3074 eukaryotic viruses. Non-CF viromes are shown in blue and CF viromes are shown in red.

Differences in eukaryotic DNA viral communities in CF versus Non-CF individuals were confirmed by PCA (Figure 5). Non-CF viromes all had nearly identical values for the first and second principal components, resulting in a tight cluster on the graph. This was largely driven by the general absence of ssDNA and Retro-transcribing viruses from Non-CF viromes (Figure 4A). Principal components for CF viromes were more variable, reflecting the tendency for CF viromes to have a small number (between one and four) of highly abundant viral species. Specifically, the outlying behavior of CF2 was driven by a high positive loading of the second principal component by the Geminivirus Sugarcane streak Egypt virus. CF4 did not cluster with other metagenomes due to a high negative loading of the first principal component by Reticuloendotheliosis virus.

In Non-CF individuals, eukaryotic viral communities likely represent transient infections rapidly cleared by immune cells or viral particles being removed from the airway via MCC. In CF individuals, communities probably correspond to more persistent infections. Viral replication is increased in the CF airway and synergism between persistent bacteria and incipient eukaryotic viruses pre-disposes CF individuals to acquiring viral infections [8]. This is in contrast to asthma, where the sequelae of viral infections are often severe in the lower respiratory tract, yet individuals are no more likely to acquire such infections [3]. It is difficult to distinguish clinical symptoms of viral infections from the typical respiratory distress associated with CF, so it is possible that CF individuals in this study could have had extant viral infections [8].

### Metabolic profiles of respiratory tract viruses

**Figure 6:**
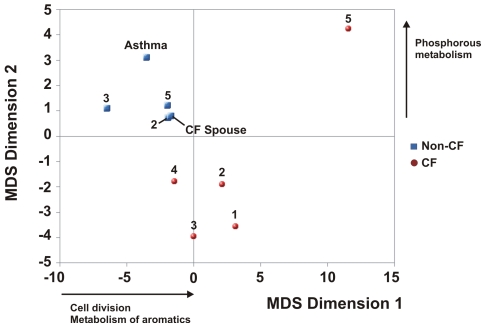
Non-metric multidimensional (NM-MDS) scaling of top-level SEED metabolic subsystems. All Non-CF metagenomes are shown in blue. CF1-5 are shown in red. The inputs to NM-MDS were the number of hits to subsystems in the highest level of the SEED hierarchy.

**Figure 7:**
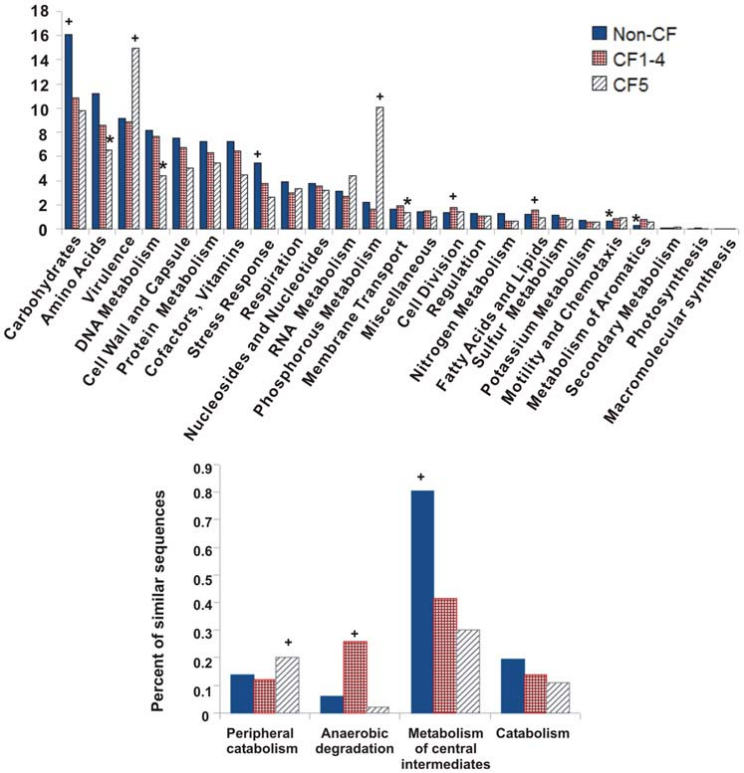
Distribution of similarities to metabolic subsystems in respiratory viromes. (A) Distribution of top-level subsystems in respiratory tract viromes. (B) Second-level subsystems from the SEED hierarchy for aromatic metabolism. Non-CF viromes are shown in blue, CF viromes 1–4 are shown in red, and CF5 is in gray. Subsystems determined by XIPE to be over-represented in a particular group are marked with a (+) while those that are under-represented are marked with an asterisk (*).

Non-CF individuals shared a common viral metabolic profile which was distinctly different from that of CF individuals (Figure 6). Functional annotations were assigned to metagenomic sequences by tBLASTx comparison to the non-redundant SEED database at the highest subsystem level, which consists of 25 classifications (Figure 7A). The percentage of known sequences (i.e., sequences with significant similarity to the database) was much higher than reported in the literature for other viral metagenomes (Figure S3) [28].

Metabolic functions encoded by viruses are determined by the environment, and functional genes carried by phage largely mirror those of their hosts [28]. The CF airway has distinct regions characterized by hypoxia and low pH, and airway secretions are enriched in amino acids, DNA, phospholipids and other cellular debris [4], [26]. The specific adaptations required for survival in this environment are reflected by the metabolic profiles of CF viromes.

Non-CF1Asthma and Non-CF4Spouse shared phage taxonomy with CF viromes, but did not share metabolic profiles because they have a Non-CF airway environment. These results are similar to findings in the human gut, where microbiomes were determined to share a set of core metabolic genes even when different microbial taxa were present, and aberrant physiological states (i.e., obesity) lead to definitive changes in the metabolic consortium [21]. As indicated by CF5, there may be more than one disease state which defines metabolism in CF, reflecting differences in pathology, disease development and/or treatment regimes.

All of the CF metagenomes (including CF5) were over-represented in functions related to the metabolism of aromatic compounds (Figure 7A). At the second hierarchical subsystem level, CF1-4 were over-represented in anaerobic degradation of aromatics, while CF5 had more genes related to peripheral catabolism pathways, most of which were aerobic (Figure 7B). Non-CF metagenomes were enriched for metabolism of central intermediates via aerobic mechanisms. CF sputum is derived from hypoxic microenvironments which require persistent microbes to acquire anaerobic adaptations [26]. Aromatic amino acids have been implicated both as preferred carbon sources and also regulators of quinolone signaling and biofilm formation for *Pseudomonas aeruginosa* in CF sputum [26], [27].

The presence of anaerobic aromatic catabolism genes in phage may represent lateral gene transfer with well-adapted hosts [46]. Alternatively, phage may be degrading aromatics in order to reduce biofilm formation and the exopolysaccharide layer, allowing access to susceptible Bacterial hosts.

CF5 was dramatically over-represented in phosphorous metabolism and virulence pathways (Figure 7A). Over 75% of tBLASTx similarities to the phosphorous metabolism subsystem were to the gene encoding Guanosine-5′-triphosphate,3′-diphosphate pyrophosphatase. This enzyme catalyzes the removal of a phosphate group from guanosine pentaphosphate (pppGpp) to generate guanosine tetraphosphate (ppGpp) [47]. Both pppGpp and ppGpp are part of the canonical bacterial stringent response which is enacted to slow growth rates during nutrient stress [48]. They have also been linked to bacterial virulence, antibiotic resistance, biofilm formation, quorum sensing, and phage induction in a variety of bacteria including *Pseudomonas aeruginosa* [47], [48]. For many bacteria ppGpp is a more potent effector molecule than pppGpp, suggesting a need for increased levels of Guanosine-5′-triphosphate,3′-diphosphate pyrophosphatase [47], [49].

### Additional considerations and recommendations for human microbiome studies

We used sputum samples as a proxy for the human respiratory tract, much as fecal samples have been used as a proxy for the human gut [18]–[20]. Expectorated sputum has been routinely exploited as a rapid, inexpensive, non-invasive method to sample the lung and lower respiratory tract, and sputum samples can achieve sensitivity and accuracy comparable to bronchoalveolar lavage for detection of respiratory infections [50]. T-RFLP analysis of bacterial communities demonstrated that sputum is not substantially contaminated by saliva and bacterial flora of the oral cavity [34]. However, the degree to which sputum represents the upper and lower respiratory tract is unknown, especially in healthy individuals. Microbial communities in fecal samples have been shown to differ significantly from those in intestinal mucosal samples, based on 16S rDNA analysis, and similarly, sputum samples may contain different communities than the lung or lower respiratory tract [19].

In this study, human genomic DNA contamination was detected bioinformatically and removed. Previously, we sequenced control viromes from CF sputum which were not DNase I treated. These metagenomes contained over 90% of sequences from human genomic DNA as determined by BLASTn analysis (data not shown). This human DNA comes from neutrophils present in the airway, either through the active dissemination of neutrophil extracellular traps (NETs) or by the release of cellular contents during cell death [51]. Using the protocol described above, the percent of human DNA detected ranged from 10% to 34% (Table 2). This was markedly lower than in the control metagenomes, and was comparable to the percentage of human DNA (24% and 36%) obtained by Allander et al. [16] for viral isolation from pooled nasal aspirate samples. As studies of the human microbiome move away from characterization of microbes using 16S rDNA and towards complete metagenomic analysis of both microbial and viral communities, human genomic DNA contamination becomes unavoidable.

**Table 2:** Characteristics of the 10 human respiratory tract viral metagenomes including GC content and CG dinucleotide relative abundance odds ratios.

Metagenome	Number of Sequences	Percent Human	Percent Non-Human	Non-Human BP	Non-Human Av Seq Length	GC Content	CG Odds Ratio
NonCF1Asthma	286192	18% (52142)	82% (234050)	54465453	229	41.3	1.07
NonCF2	281687	34% (97091)	66% (184596)	41522972	215	40.1	1.01
NonCF3	240848	10% (23149)	90% (217699)	51487425	236	40.5	0.98
NonCF4Spouse	339107	32% (107911)	68% (231196)	53415637	232	40.6	0.89
NonCF5	345112	18% (62232)	82% (282880)	62464670	219	43.5	0.97
CF1	180647	19% (33451)	81% (147196)	33631380	226	41.5	1.04
CF2	225240	15% (33594)	85% (191646)	43741746	228	43.3	1.00
CF3	184410	25% (45891)	75% (138519)	34335377	246	42.8	0.87
CF4	266135	18% (28593)	82% (217270)	45559961	203	43.2	0.84
CF5	220356	18% (39909)	82% (180447)	41011286	226	43.0	1.09

All odds ratios were within normal range (0.78 to 1.23). All human sequences were removed prior to further bioinformatic analysis.

After all contaminating sequences were removed, there were still at least 130,000 sequences comprising over 30 Mbp in all metagenomes. To verify the presence of viruses in the metagenomes, we assembled two metagenomes and compared contigs to the non-redudant database using BLASTn. There were 23 contigs assembled from Non-CF2 which had BLASTn matches to *Streptococcus* phage Cp-1 (E-value <10^−5^), with an alignment length greater than 50 bp, and greater than 85% identity (Figure S4). The assembly of the CF3 metagenome yielded high coverage and significant BLASTn hits to the genome of *H. influenza* prophage Mu (Figure S5).

Here, we isolated DNA viruses from sputum, including both phage and eukaryotic viruses. The majority of respiratory infections (>75%) have been attributed to RNA viruses such as rhinoviruses, coronaviruses, and paramyxoviruses, so many previous studies have focused on the characterization of RNA viruses in the respiratory tract [38]. CF is predominantly a microbial disease, and phage are known to exert important top-down controls on microbial communities [52]. However, little work has been done to describe phage communities and DNA viruses associated with CF or with the airways in general [4], [38]. Over 98% of all completely sequenced phage have DNA genomes, therefore to assess phage diversity, taxonomy, and function, it was necessary to isolate viral DNA [53]. Future studies of the respiratory tract virome should be expanded to include characterization of RNA viral communities.

A caveat to this study was the use of Multiple Displacement Amplification (MDA) with phi29 polymerase to amplify viral DNA prior to pyrosequencing. MDA generally provides an even representation of genomes except at the ends, however, certain genomes (small and circular or large and linear) may be preferentially amplified [54], [55]. To avoid random biases introduced by initial reaction conditions, we performed five separate amplifications which were then combined. All of the metagenomes used here were collected, processed and amplified in an identical manner, so any biases would have been introduced equally in all samples.

### Conclusions

Metagenomic analysis of the human respiratory tract DNA virome illustrated that airway viral communities in the diseased and non-diseased states are defined by metabolism and not by taxonomy. The non-diseased airway virome contains a set of shared core metabolic functions, which deviate strongly in the face of chronic disease. These deviations are driven by dramatic environmental changes in the airways, induced by the nature of cystic fibrosis, such as the introduction of hypoxic microenvironments and novel carbon sources [26], [27]. In cases where phage taxonomy was shared between Non-CF and CF individuals, metabolic functions still remained distinct. The converse was also true, that is, even when Non-CF viromes differed in phage and eukaryotic viral constituents, they maintained typical Non-CF metabolic profiles. The presence of two alternative metabolic states in CF reflects the heterogenous nature of disease. Though CF is generally considered to be well-characterized, there is still inherent individual variation. The need for alternative therapies for CF is increasing, as microbial antibiotic resistance becomes widespread. The results of this study suggest that CF therapeutics might be better aimed at changing the environment of the airways rather than targeting dominant taxa.

## Methods

### Ethics statement

Subject recruitment and sample collection were approved by the San Diego State University Institutional Review Board (SDSU IRB 2121) and Environmental Health Services (BUA 06-02-062R). Written consent forms were obtained from all study subjects.

### Study population

The five individuals with CF who volunteered for this study were patients at the Cystic Fibrosis Foundation accredited Adult cystic fibrosis Clinic at the University of California San Diego Medical Center. Patients were eligible if they could be classified as clinically stable (i.e., in a non-exacerbated state and free from systemic antibiotic therapy for at least thirty days), and had no reportable cold or flu-like symptoms in the previous thirty days. All volunteers with CF were screened for signs and symptoms of a upper respiratory infection for the thirty days prior to the study. All CF subjects were required to have a well documented diagnosis with either two known mutations in the cystic fibrosis Transmembrane Regulator (CFTR) or an abnormally high sweat chloride test. In addition, all CF patients had *Pseudomonas aeruginosa* present in their sputum, as determined by culturing in the clinic's microbiology lab. The five CF individuals randomly selected for the study consisted of two males and three females. The age range was from 20 to 35 years and all patients had severe airway obstruction as assessed by standard spirometry (FEV1<50% of predicted).

Four Non-CF volunteers were recruited from the campus of San Diego State University, and were subject to the same exclusion criteria for upper respiratory infection. One of these Non-CF individuals had mild asthma controlled by medication. A final Non-CF volunteer was the spouse of a CF patient and was recruited from the greater San Diego area. The five Non-CF individuals consisted of four females and one male, with an age range of 24 to 50 years.

### Sample collection

Sputum samples of approximately 10 ml were obtained from CF patients at the Adult cystic fibrosis Clinic by expectoration into a sterile cup, as directed by clinic staff. Since sputum expectoration is difficult in general for Non-CF individuals, all Non-CF subjects were first required to do an oral rinse with water to prevent excessive salivary contamination and then take five deep breaths to loosen lung secretions. Subjects were then instructed to cough deeply into a sterile cup. The deep breathing and coughing procedures were repeated until at least 1 ml of sputum was obtained.

### Metagenomic library preparation

All sputum samples were diluted with an equal volume of Suspension Medium (SM) buffer (1 M NaCl, 10 mM MgSO_4_, 50 mM Tris-HCl pH 7.4). To aid in mucus dissolution, samples were incubated with 10 ml of 6.5 mM dithiothreitol (Acros Organics: Morris Plains, New Jersey) for 30 minutes at 37°C. The treated sputum was homogenized using a PowerGen 125 mechanical homogenizer (Fisher Scientific: Hampton, New Hampshire) until it was uniform in color and there was no visible particulate debris. Homogenized samples were filtered through a 0.8 micron black polycarbonate filter (GE Water & Process Technologies: Trevose, Pennsylvania) followed by a 0.45 micron MILLEX®HV filter (Millipore: Carrigtwohill, Colorado) to remove eukaryotic and microbial cells. Viruses in the 0.45 micron filtrate were purified and concentrated using a cesium chloride (CsCl) gradient to remove free DNA and any remaining cellular material [56]. After collection of viral concentrates from the CsCl gradient, the presence of virus-like particles (VLPs) and the absence of microbial contamination were verified by epifluorescence microscopy using SYBR® Gold (Invitrogen: Eugene, Oregon) as described in [56]. Sputum samples from healthy subjects contained approximately 10^7^ VLPs per ml, while the samples from CF patients contained approximately 10^9^ VLPs per ml. A sample epifluoresence micrograph is shown in Figure S6. Chloroform was added to the viral concentrates to rupture the membranes of any remaining cells. Following a one hour incubation and centrifugation, choloroform was removed by pipetting. To degrade any remaining free DNA prior to viral DNA extraction, samples were treated with 2 units per µl of Dnase I (Sigma-Aldrich: St. Louis, MO) at 37°C for 1 hour. Viral DNA was isolated using CTAB/phenol:choloroform extractions and amplified using multiple displacement amplification with Phi29 polymerase [56]. Viral DNA was sequenced at 454 Life Sciences (Branford, CT) using the GSFLX pyrosequencing platform to produce ten total viral metagenomic libraries. The ten viral metagenomes are accessible from NCBI (www.ncbi.nlm.gov) under the genome project ID 39545.

### Initial bioinformatic processing of metagenomes

All metagenomes were compared to the Human Genome build 36.3 (http://www.ncbi.nlm.mih.gov) using BLASTn to determine how effective the combination of cesium chloride density gradient centrifugation and DNase I treatment was for removing human genomic contamination from the viral preps [39]. Sequences with 80% identity over 80% of their length to human sequences were considered contaminating human genomic DNA and were removed prior to further bioinformatic analyses. Characteristics of viromes and the percentage of human genomic sequences detected are provided in Table 2.

Following removal of human sequences, dinucleotide relative abundance analysis was used as a secondary screen to detect human DNA contamination, which manifests as an overall depression of CG dinucleotides [57], [58]. In all of the decontaminated metagenomes, the relative abundance odds ratios for CG dinucleotides were between 0.83 and 1.09, within the normal range as defined by Karlin, indicating successful removal of human DNA (Table 2) [57], [58]. All viromes were AT rich (in comparison to microbial metagenomes) as expected, with GC content between 40–43%, just below the average of approximately 45% previously reported for viral metagenomes [58]. The human genomic DNA decontaminated metagenomic libraries were named according to the subject group they were derived from (Non-CF or CF) and were numbered 1 through 5 in each group. Viromes derived from the individual with asthma and the CF spouse were designated as Non-CF1Asthma and Non-CF4Spouse.

### Diversity estimation

To estimate viral diversity and community structure within metagenomes, contig spectra were generated using the free software Circonspect (http://sourceforge.net/projects/circonspect/). Average contig spectra were calculated using assemblies of 10,000 randomly selected sequences with enough repetitions to achieve 2× coverage of each metagenome. The assembly parameters were 98% minimal match and 35 base pair overlap. Sequences less than 100 base pairs were discarded and all other sequences were trimmed to 100 base pairs prior to assembly to obtain identical sequence size in the repeated assemblies. Average contig spectra were used as inputs to Phage Communities from Contig Spectra (PHACCS) tool (http:biome.sdsu.edu/phaccs), which estimates diversity using rank-abun [36]. Diversity estimates were based on the best-fit model, in this case the logarithmic model.

### Sequential BLAST analysis

Metagenomic libraries were compared to each other using BLASTn to find shared sequences between all Non-CF viromes and all CF viromes. One metagenome from each set (Non-CF or CF) was chosen randomly and compared to a second randomly selected metagenome. Common sequences (E-value<10^−5^ and a minimum of 98% similarity over at least 35 base pairs) were identified and then used as a database for BLASTn versus a third metagenome. This was repeated for the fourth and fifth metagenomes. The entire process was repeated using a different random ordering of metagenomes. Sequential BLASTn analysis resulted in two datasets, one containing sequences common to all Non-CF metagenomes and the other with sequences common to CF metagenomes. The common Non-CF sequences were then compared using BLASTn to all CF metagenomes to determine which sequences were not present in any CF library (i.e., unique to Non-CF individuals). This was also performed in reverse, to find unique CF sequences.

### Comparison to phage and viral genome databases

Metagenomic libraries were compared to two boutique databases, the first containing 510 complete phage genomes (http://phage.sdsu.edu/phage) and the second, 3,074 complete eukaryotic viral genomes (http://www.ncbi.nlm.nih.gov/genomes/VIRUSES/viruses.html) using tBLASTx with an E-value cutoff of 10^−5^ [39]. Counts of best tBLASTx similarities to each genome were normalized for genome size by weighting the number of significant similarities by the total number of base pairs in the database divided by the size of the genome in base pairs. Similarity counts were also normalized for the number of sequences per metagenome, to allow direct comparisons between metagenomes. Normalized best tBLASTx similarities to the phage database were plotted against the Phage Proteomic Tree version 4 (http://phage.sdsu.edu/~rob/PhageTree/v4) using Bio-Metamapper [53], [56]. Similarities to dsDNA, ssDNA, and retro-transcribing eukaryotic viruses were plotted according to NCBI taxonomy (http://www.ncbi.nlm.nih.gov/genome). Similarities to RNA viruses were not included because they were artifactual, since only DNA was sequenced in this study. Significant similarities to RNA viruses comprised less than 1% of all tBLASTx similarities.

### Assessment of metabolic potential

The metabolic potential of each virome was assessed by BLASTx (E-value<10^−5^) comparison to the SEED database using the MG-RAST service [59], [60]. MG-RAST assigns sequences to three hierarchical levels of metabolic subsystems, which consist of groups of genes that comprise a metabolic function or pathway [61]. The non-parametric statisical program XIPE was used to detect significant differences between metabolic profiles of viral metagenomes at a 95% confidence level [62]. XIPE identifies the specific subsystems driving the differences between metagenomes, and in which metagenome the function was are over-represented.

### Complete metagenomic assembly

Complete assembly of the Non-CF2 and CF3 metagenomes was performed using PHRAP as a quality check to confirm sucessful isolation of viral genomes [63]. These two metagenomes were assembled because they had high coverage of phage genomes as indicated by tBLASTx. There were 9,508 contigs ranging in size from 40 to 14,982 bp for Non-CF2, and 8,163 contigs from 212 to 7,748 base pairs for CF3. Contigs were compared to the non-redundant nucleotide database maintained at NCBI (http://www.ncbi.nlm.nih.gov) using BLASTn to assign taxonomy.

### Statistical analyses

All statistical analyses, with the exception of XIPE, were performed using the software package R (www.r-project.org) [64]. Principal components analysis (PCA) with the R function *prcomp* was used to examine overall taxonomic similarities between metagenomes [65]. The first two principal components were used to generate 2D scatter plots. Non-metric multidimensional scaling (NM-MDS) with the R function *isoMDS* was used to determine relationships between metagenomes based on metabolic profiles. The analysis was performed with NM-MDS instead of PCA for metabolic potential because all metagenomes had at least one hit to each of the 25 subsystems (i.e., there were no zero values). Similar to PCA, NM-MDS does not require *a priori* classification of the data and plotting the MDS coordinates shows natural grouping patterns. Clusters observed in PCA and NM-MDS scatterplots were confirmed statistically using k-means clustering. To determine the optimal number of clusters, within-group sums of squares were calculated for partitions involving between 1 and 9 clusters [63], [65]. Cluster membership was determined by using the R function *kmeans* with the optimal number of clusters.

## Supporting Information

Table S1 Taxonomic designations of metagenomic sequences based on BLASTn (e-value<10−5) comparison to the non-redundant database at NCBI. Sequences which had no significant similarities were assigned as “unknown”, while those with significant similarities were considered to be “known”. There were no sequences with significant similarities to Archaea, and therefore known sequences were classified as either viral (including phage and eukaryotic viruses), bacterial, or eukaryotic.
(0.02 MB DOC)

Table S2 Results of comparison of metagenomes to the database of 510 fully sequenced phage genomes using tBLASTx (e-value<10−5). The number of unique genomes refers to how many phage genomes had a significant BLAST similarity in only one of the five Non-CF or CF metagenomes.
(0.02 MB DOC)

Table S3 Relative abundances of the 19 phage genomes which appear in all human respiratory tract viromes based on tBLASTx similarities (e-value<10−5). Relative abundances were calculated as the normalized number of similarities to each phage divided by the total number of similarities to phage for each metagenome.
(0.02 MB DOC)

Table S4 Results of comparison of metagenomes to the database of 3074 fully sequenced eukaryotic viral genomes using tBLASTx (e-value<10−5). The number of unique genomes refers to how many viral genomes had a significant BLAST similarity in only one of the five Non-CF or CF metagenomes.
(0.02 MB DOC)

Table S5 Relative abundances of the 20 eukaryotic DNA viral genomes which appear in all human respiratory tract viromes based on tBLASTx similarities (e-value<10−5). Relative abundances were calculated as the normalized number of similarities to each virus divided by the total number of similarities to eukaryotic DNA viruses for each metagenome.
(0.02 MB DOC)

Figure S1 Figure S1. Combined coverage of Retiucloendotheliosis virus across all CF metagenomes as determined by tBLASTx. The graphic of the 8295 kb Reticuloendotheliosis genome is from NCBI (http://www.ncbi.nlm.nih.gov).
(0.03 MB PNG)

Figure S2 Supplementary Figure 2. Combined coverage of Suid herpesvirus 1 and Cercopithecine herpesvirus 2 across all Non-CF and CF metagenomes as determined by tBLASTx. The graphics of the two reference genomes are from NCBI (http://www.ncbi.nlm.nih.gov).
(0.11 MB PNG)

Figure S3 Supplementary Figure 3. Percentage of metagenomic sequences with known and unknown metabolic functions as determined by BLASTx to the SEED database. A sequence was considered as known if it had a significant (e-value<10−5) hit to a gene in a metabolic pathway.
(0.02 MB PNG)

Figure S4 Supplementary Figure 4. Coverage of the Streptococcus pneumonia phage Cp-1 genome in metagenome Non-CF2 by raw metagenomic sequences as determined by tBLASTx (A) and by assembled contigs as determined by BLASTn (B). The graphic of the 19,343 kb phage Cp-1 genome is from NCBI (http://www.ncbi.nlm.nih.gov).
(0.11 MB PDF)

Figure S5 Coverage of the Haemophilus influenza prophage Mu genome in the CF6 metagnome by raw metagenomic sequences as determined by TBLASTX (A) and by assembled contigs as determined by BLASTn (B). The graphic of the 43033 kb prophage Mu genome is from NCBI (http://www.ncbi.nlm.nih.gov).
(0.21 MB PDF)

Figure S6 Virus-like Particles (VLPs) from the sputum sample of a CF patient. The VLPs were visualized by capture on a 0.02 µm Anodisc filter, SYBR Gold staining, and viewing under an epifluorescence microscope. The viruses appear as tiny bright pinpricks of light.
(0.26 MB PDF)
