# Supplementary figures and images for: Metagenomic Analysis of Respiratory Tract DNA Viral Communities in Cystic Fibrosis and Non-Cystic Fibrosis Individuals
Source: PLoS One. 2009 Oct 9;4(10):e7370. doi: 10.1371/journal.pone.0007370 (PMC2756586; doi:10.1371/journal.pone.0007370)

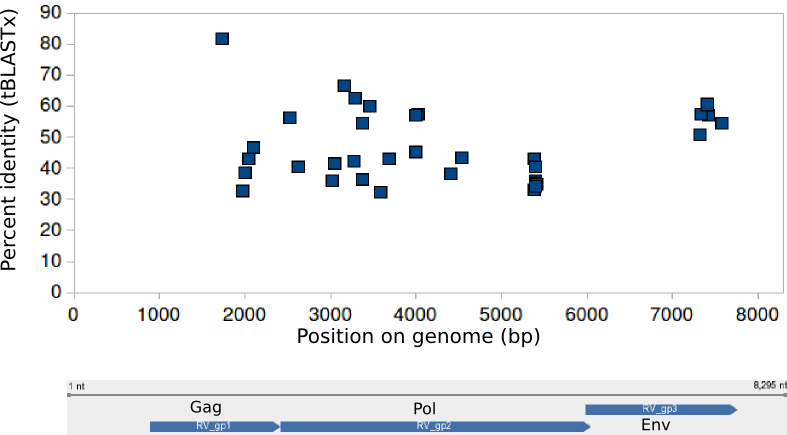

Supplement: Figure S1 — Combined coverage of Retiucloendotheliosis virus across all CF metagenomes as determined by tBLASTx. The graphic of the 8295 kb Reticuloendotheliosis genome is from NCBI (http://www.ncbi.nlm.nih.gov). (0.03 MB PNG) [file pone.0007370.s006.png]

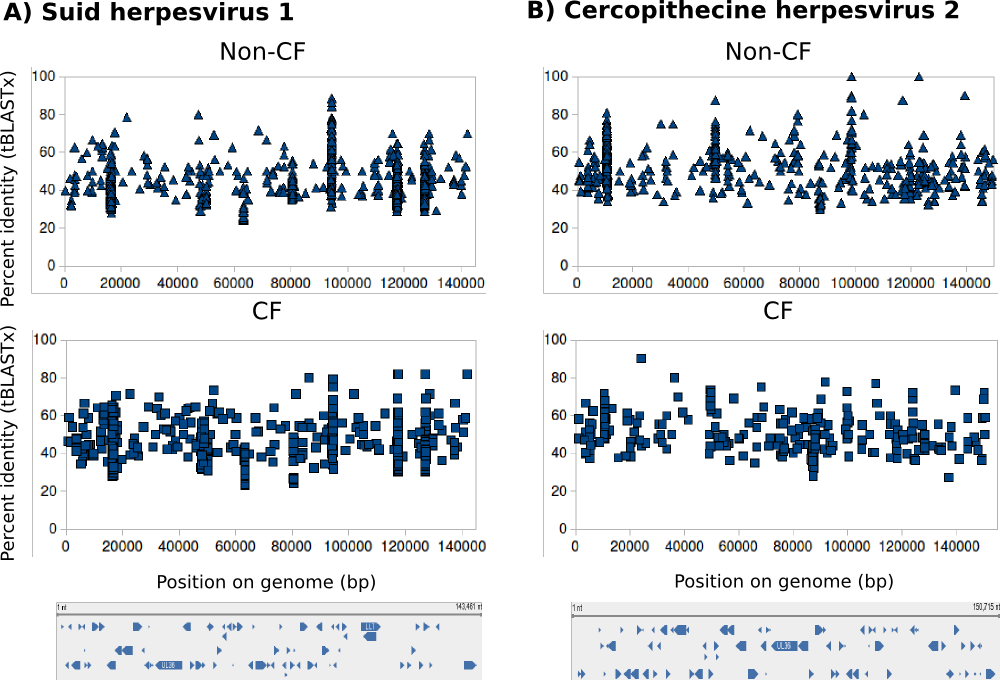

Supplement: Figure S2 — Supplementary Figure 2. Combined coverage of Suid herpesvirus 1 and Cercopithecine herpesvirus 2 across all Non-CF and CF metagenomes as determined by tBLASTx. The graphics of the two reference genomes are from NCBI (http://www.ncbi.nlm.nih.gov). (0.11 MB PNG) [file pone.0007370.s007.png]

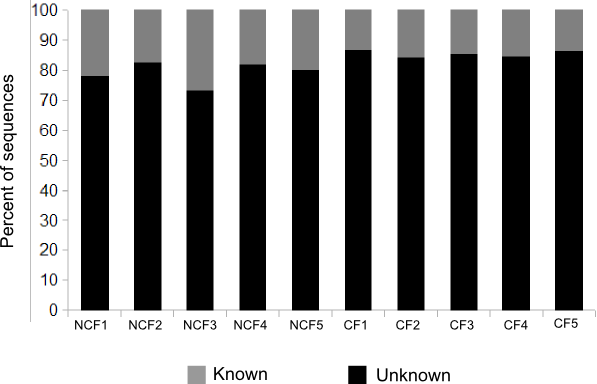

Supplement: Figure S3 — Supplementary Figure 3. Percentage of metagenomic sequences with known and unknown metabolic functions as determined by BLASTx to the SEED database. A sequence was considered as known if it had a significant (e-value<10−5) hit to a gene in a metabolic pathway. (0.02 MB PNG) [file pone.0007370.s008.png]

**A**

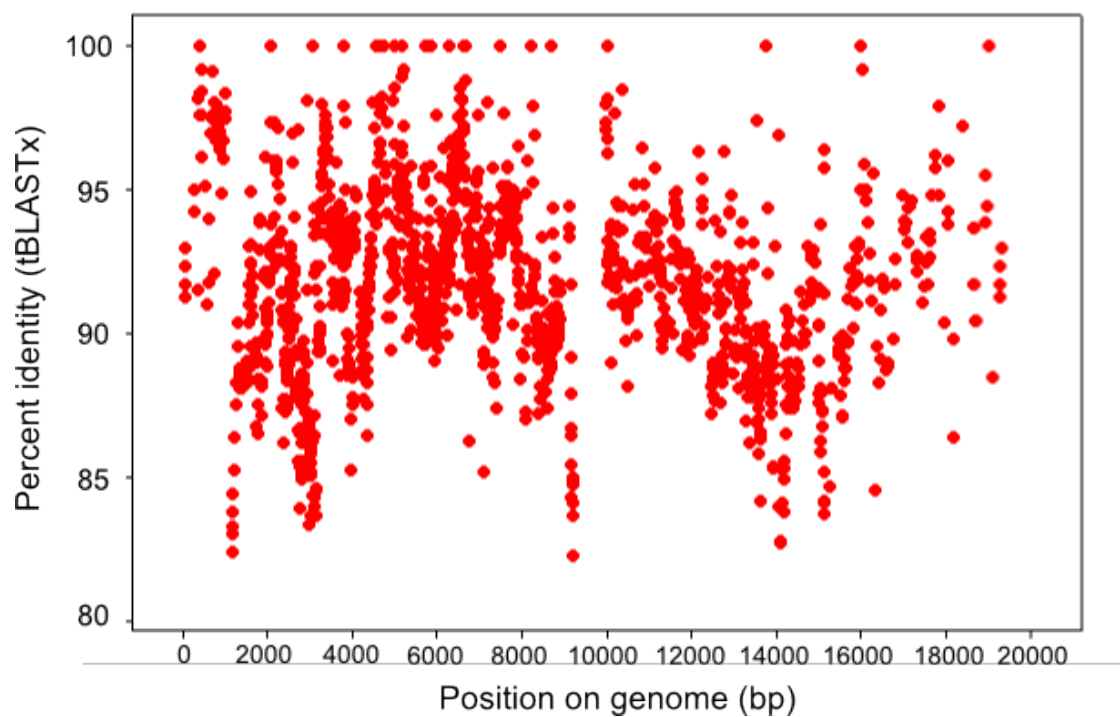

**B**

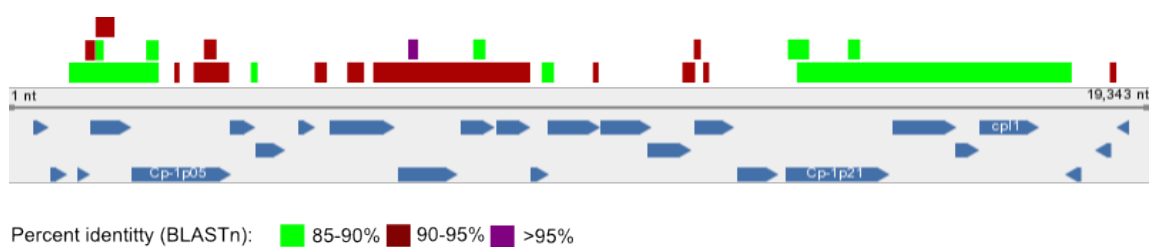

Supplement: Figure S4 — Supplementary Figure 4. Coverage of the Streptococcus pneumonia phage Cp-1 genome in metagenome Non-CF2 by raw metagenomic sequences as determined by tBLASTx (A) and by assembled contigs as determined by BLASTn (B). The graphic of the 19,343 kb phage Cp-1 genome is from NCBI (http://www.ncbi.nlm.nih.gov). (0.11 MB PDF) [file pone.0007370.s009.pdf]

A

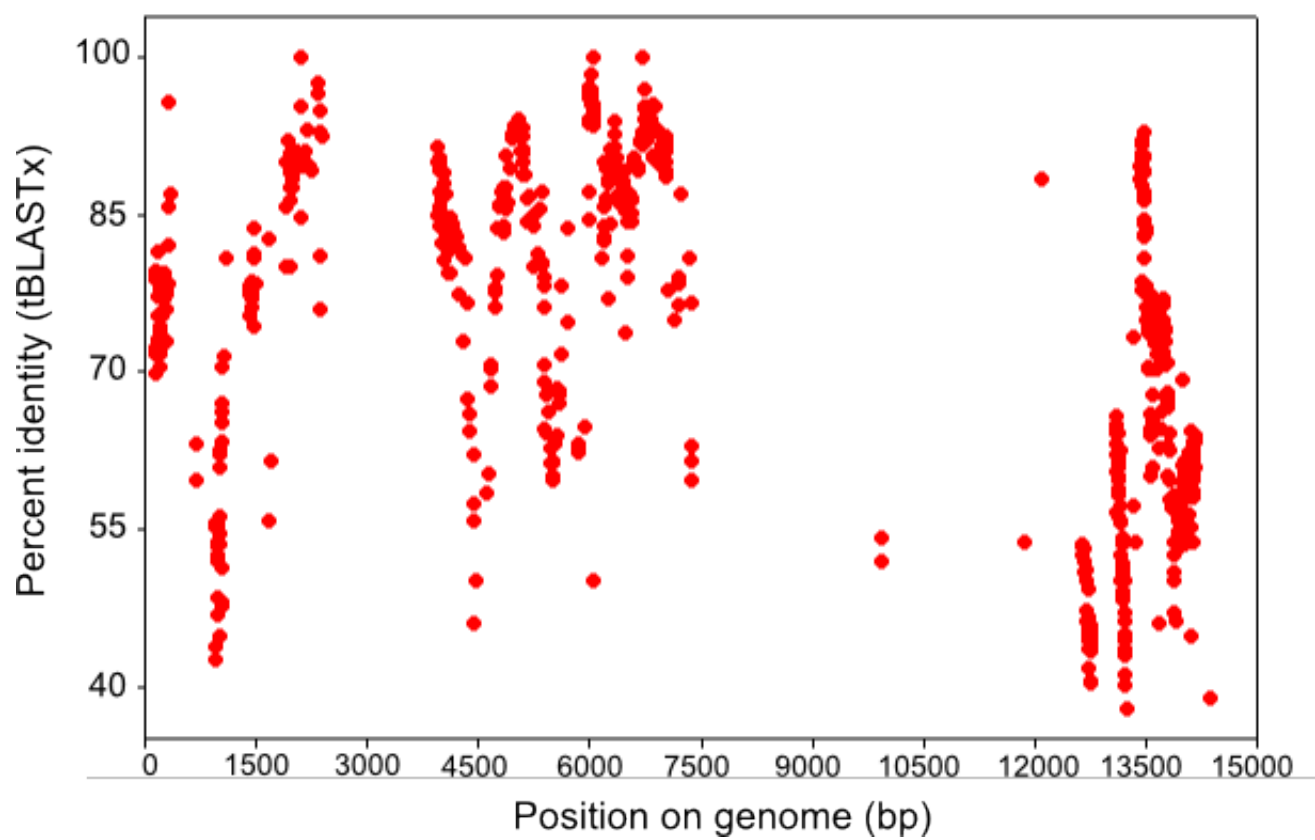

B

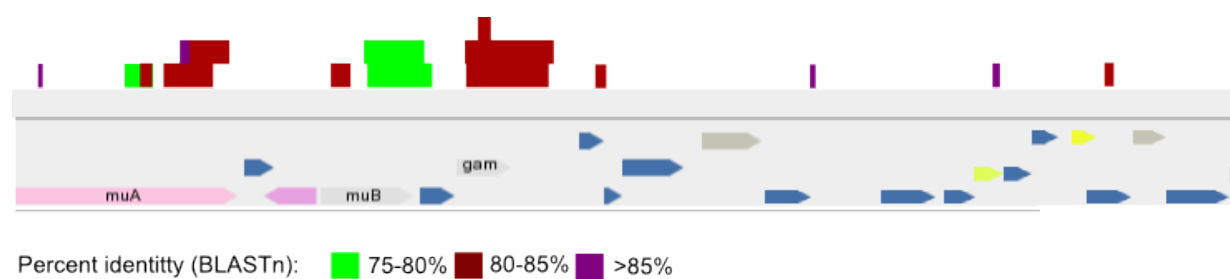

Supplement: Figure S5 — Coverage of the Haemophilus influenza prophage Mu genome in the CF6 metagnome by raw metagenomic sequences as determined by TBLASTX (A) and by assembled contigs as determined by BLASTn (B). The graphic of the 43033 kb prophage Mu genome is from NCBI (http://www.ncbi.nlm.nih.gov). (0.21 MB PDF) [file pone.0007370.s010.pdf]

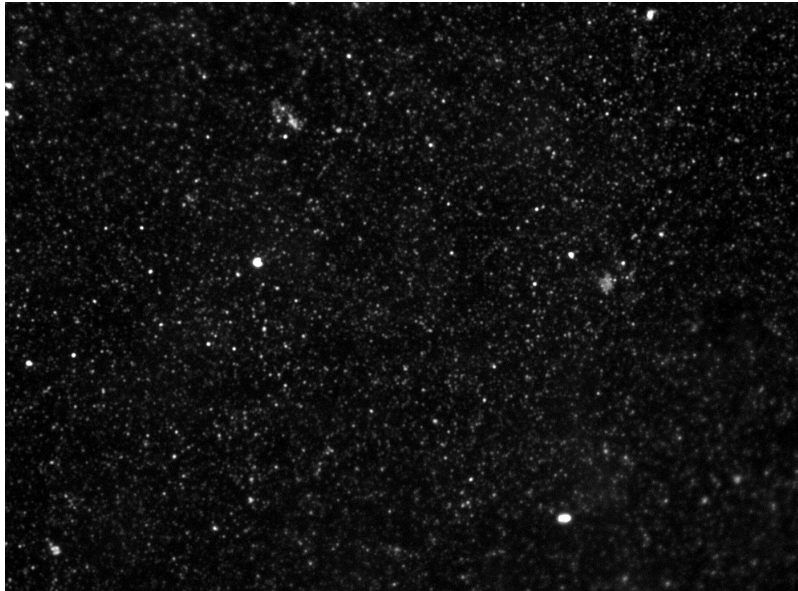

Supplement: Figure S6 — Virus-like Particles (VLPs) from the sputum sample of a CF patient. The VLPs were visualized by capture on a 0.02 µm Anodisc filter, SYBR Gold staining, and viewing under an epifluorescence microscope. The viruses appear as tiny bright pinpricks of light. (0.26 MB PDF) [file pone.0007370.s011.pdf]
