# Supplementary material for: Metagenomic Analysis of Respiratory Tract DNA Viral Communities in Cystic Fibrosis and Non-Cystic Fibrosis Individuals
Source: PLoS One. 2009 Oct 9;4(10):e7370. doi: 10.1371/journal.pone.0007370 (PMC2756586; doi:10.1371/journal.pone.0007370)
Supplement: Supplementary file 2 [file pone.0007370.s001.doc]

Library Name	Known, %	Viral, %	Bacterial, %	Eukaryotic, %
Non-CF1Asthma	4.76	0.30	99.46	0.01
Non-CF2	2.80	25.11	72.88	0.01
Non-CF3	7.89	0.05	99.64	0.01
Non-CF4Spouse	4.12	0.10	99.42	0.01
Non-CF5	4.20	0.06	98.63	0.01
**Non-CF Average**	4.75	5.12	94.01	0.01
CF1	8.44	0.02	99.73	0.01
CF2	6.40	0.11	99.57	0.01
CF3	7.61	6.24	93.65	<0.01
CF4	3.91	0.09	99.15	0.02
CF5	9.12	1.28	98.67	0.01
