# Supplementary material for: Metagenomic Analysis of Respiratory Tract DNA Viral Communities in Cystic Fibrosis and Non-Cystic Fibrosis Individuals
Source: PLoS One. 2009 Oct 9;4(10):e7370. doi: 10.1371/journal.pone.0007370 (PMC2756586; doi:10.1371/journal.pone.0007370)
Supplement: Supplementary file 3 [file pone.0007370.s002.doc]

	Number of hits (% of metagenome)	Number of genomes hit (% of database)	Number of unique genomes (% of genomes hit)
Non-CF1Asthma	5754 (2.38%)	220 (43.14%)	20 (9.09%)
Non-CF2	2020 (0.98%)	72 (14.12%)	6 (8.33%)
Non-CF3	2906 (1.29%)	73 (14.31%)	5 (6.85%)
Non-CF4Spouse	4851 (1.97%)	241 (47.25%)	31 (12.86%)
Non-CF5	782 (0.25%)	109 (21.37%)	15 (13.76%)
CF1	6888 (4.27%)	234 (45.88%)	4 (1.71%)
CF2	1192 (0.55%)	164 (32.16%)	3 (1.83%)
CF3	22365 (14.42%)	321 (62.94%)	13 (4.05%)
CF4	4198 (1.77%)	298 (58.43%)	16 (5.37%)
CF5	20264 (10.16%)	366 (71.76%)	34 (9.29%)
