# Supplementary material for: Metagenomic Analysis of Respiratory Tract DNA Viral Communities in Cystic Fibrosis and Non-Cystic Fibrosis Individuals
Source: PLoS One. 2009 Oct 9;4(10):e7370. doi: 10.1371/journal.pone.0007370 (PMC2756586; doi:10.1371/journal.pone.0007370)
Supplement: Supplementary file 4 [file pone.0007370.s003.doc]

	Non-CF1 Asthma	Non-CF2	Non-CF3	Non-CF4 Spouse	Non-CF5	CF1	CF2	CF3	CF4	CF5
Aeromonas hydrophila phi Aeh1	0.05	0.01	0.05	0.06	0.04	0.02	0.05	0.01	0.03	0.04
Aeromonas phi 31	0.80	0.01	0.03	0.38	0.12	0.19	0.12	0.06	0.22	0.02
Bacillus cereus phi phBC6A51	0.02	0.22	0.09	0.15	0.07	0.08	0.12	0.13	0.28	0.05
Bacillus subtilis phi 105	0.20	0.16	0.03	0.12	13.43	0.20	0.09	0.04	0.04	0.02
Bacillus subtilis phi SPBc2	0.19	0.01	0.01	0.32	0.03	0.34	0.75	0.05	0.30	0.02
Brucella melitensis 16M phi Bruc1 prophage	8.96	0.05	59.40	7.04	1.00	3.65	5.54	0.54	5.24	0.48
Escherichia coli phi CP073-4 prophage	0.70	0.06	0.10	0.58	0.15	0.18	1.43	0.29	0.41	0.06
Escherichia coli phi CP4-6 prophage	6.13	0.09	0.50	4.40	4.72	4.13	2.61	0.65	2.85	0.68
Escherichia coli phi QIN prophage	9.11	0.05	0.20	0.04	2.54	0.25	0.18	0.01	0.47	0.06
Escherichia coli phi Sp18 prophage	2.54	0.12	1.49	1.86	4.98	1.25	1.55	0.22	1.32	0.22
Haemophilus influenzae phi HP1	0.11	3.25	21.06	15.12	0.39	14.26	2.11	0.74	0.05	1.25
Lactobacillus plantarum phi LP65	0.07	0.01	0.10	0.16	0.03	0.44	0.03	0.01	0.22	0.01
Mycobacterium phi Bxz1	0.69	0.07	0.13	0.40	0.40	1.17	0.55	0.05	0.21	0.19
Mycobacterium phi CJW1	0.19	0.04	0.07	0.52	1.42	0.10	0.47	0.09	0.49	0.16
Pseudomonas phi KZ	0.23	0.05	0.09	0.03	0.37	0.20	0.08	0.02	0.10	0.36
Shigella flexneri phi Flex4 prophage	0.36	0.03	1.81	0.61	5.28	0.14	0.44	0.12	0.76	0.06
Staphylococcus phi Twort	0.07	0.01	0.01	0.07	0.06	0.29	0.14	0.02	0.12	0.07
Vibrio parahaemolyticus phi KVP40	0.18	0.03	0.07	0.17	0.08	0.05	0.18	0.02	0.18	0.10
Xylella fastidiosa phi Xpd5 prophage	2.19	0.16	0.17	2.06	1.15	1.56	1.71	0.31	1.32	0.33
