# Supplementary material for: Metagenomic Analysis of Respiratory Tract DNA Viral Communities in Cystic Fibrosis and Non-Cystic Fibrosis Individuals
Source: PLoS One. 2009 Oct 9;4(10):e7370. doi: 10.1371/journal.pone.0007370 (PMC2756586; doi:10.1371/journal.pone.0007370)
Supplement: Supplementary file 5 [file pone.0007370.s004.doc]

	Number of hits (% of metagenome)	Number of genomes hit (% of database)	Number of unique genomes (% of genomes hit)
Non-CF1Asthma	4935 (2.04%)	113 (4.44%)	7 (6.19%)
Non-CF2	4881 (2.38%)	107 (4.20%)	9 (8.41%)
Non-CF3	3125 (1.38%)	101 (3.97%)	4 (3.96%)
Non-CF4Spouse	65 (0.03%)	29 (1.14%)	1 (3.45%)
Non-CF5	6122 (1.99%)	125 (4.91%)	12 (9.60%)
CF1	1984 (1.23%)	99 (3.89%)	24 (24.2%)
CF2	1895 (0.87%)	79 (3.10%)	7 (7.59%)
CF3	1687 (1.09%)	87 (3.42%)	5 (5.75%)
CF4	3253 (1.37%)	110 (4.32%)	26 (23.64%)
