# Supplementary material for: Metagenomic Analysis of Respiratory Tract DNA Viral Communities in Cystic Fibrosis and Non-Cystic Fibrosis Individuals
Source: PLoS One. 2009 Oct 9;4(10):e7370. doi: 10.1371/journal.pone.0007370 (PMC2756586; doi:10.1371/journal.pone.0007370)
Supplement: Supplementary file 6 [file pone.0007370.s005.doc]

	Non-CF1 Asthma	Non-CF2	Non-CF3	Non-CF4 Spouse	Non-CF5	CF1	CF2	CF3	CF4	CF5
Acanthamoeba polyphaga mimivirus	0.91	0.14	2.83	0.38	0.92	1.17	2.47	0.33	0.19	0.26
Aedes taeniorhynchus iridescent virus	0.31	0.03	0.08	0.29	0.04	0.16	0.15	0.02	0.03	0.03
Amsacta moorei entomopoxvirus 'L'	1.01	0.15	2.61	1.44	0.79	1.77	2.06	0.27	0.15	0.18
Bovine adenovirus A	0.12	0.06	0.84	0.01	0.18	0.29	0.67	0.32	0.12	0.30
Bovine herpesvirus 5	0.60	0.04	0.98	1.22	0.51	0.29	0.25	0.11	0.04	1.18
Cercopithecine herpesvirus 1	0.50	0.04	0.49	1.42	0.39	0.19	0.11	0.05	0.02	1.04
Cercopithecine herpesvirus 16	0.92	0.09	1.38	0.36	2.73	1.08	1.27	0.29	0.10	1.60
Cercopithecine herpesvirus 2	0.65	0.04	1.05	0.37	2.76	0.92	0.87	0.30	0.04	1.73
Cercopithecine herpesvirus 9	0.07	0.02	2.73	0.45	0.09	0.16	0.18	0.03	0.00	0.02
Chlorella virus ATCV-1	3.40	0.51	7.69	0.58	1.81	4.06	4.31	0.90	0.61	0.61
Chlorella virus FR483	0.71	0.19	2.33	0.35	0.59	0.96	3.97	0.27	0.32	0.31
Ectocarpus siliculosus virus 1	0.83	0.12	2.72	0.33	0.49	0.63	1.07	0.34	0.24	0.14
Frog virus 3	0.04	0.09	0.59	0.53	0.08	0.51	0.70	0.01	0.02	0.16
Human herpesvirus 1	0.29	0.56	0.50	1.83	1.21	0.39	0.19	0.08	0.18	0.90
Human herpesvirus 2	0.42	0.01	0.22	0.00	0.92	0.64	0.66	0.22	0.05	0.60
Melanoplus sanguinipes entomopoxvirus	0.09	0.02	0.69	0.24	0.08	0.04	0.36	0.05	0.05	0.04
Paramecium bursaria Chlorella virus AR158	0.83	0.19	2.63	0.81	0.76	1.85	3.31	0.46	0.30	0.31
Suid herpesvirus 1	1.40	0.38	2.88	1.56	1.68	0.83	0.75	0.23	0.12	1.84
Taterapox virus	0.59	0.09	0.05	0.28	0.18	0.08	0.09	0.01	0.09	0.06
